# *Leishmania (Viannia) braziliensis* Inositol Phosphorylceramide: Distinctive Sphingoid Base Composition

**DOI:** 10.3389/fmicb.2017.01453

**Published:** 2017-08-04

**Authors:** Erica V. De Castro Levatti, Marcos S. Toledo, Renata Watanabe Costa, Diana Bahia, Renato A. Mortara, Helio K. Takahashi, Anita H. Straus

**Affiliations:** ^1^Departamento de Bioquímica, Escola Paulista de Medicina, Universidade Federal de São Paulo São Paulo, Brazil; ^2^Departmento de Microbiologia, Imunologia e Parasitologia, Escola Paulista de Medicina, Universidade Federal de São Paulo São Paulo, Brazil; ^3^Departamento de Biologia Geral, Instituto de Ciências Biológicas, Universidade Federal de Minas Gerais Belo Horizonte, Brazil

**Keywords:** antibody, ceramide, phosphoinositide, mass spectrometry, sphingolipid, eicosasphinganine, eicosasphingosine

## Abstract

Inositol phosphorylceramide (IPC), the major sphingolipid in the genus *Leishmania* but not found in mammals, is considered a potentially useful target for chemotherapy against leishmaniasis. *Leishmania (Viannia) braziliensis* is endemic in Latin America and causes American tegumentary leishmaniasis. We demonstrated that IPCs are localized internally in parasites, using a specific monoclonal antibody. Treatment with 5 μM myriocin (a serine palmitoyltransferase inhibitor) rendered promastigotes 8-fold less infective than controls in experimental hamster infection, as determined by number of parasites per inguinal lymph node after 8 weeks infection, suggesting the importance of parasite IPC or sphingolipid derivatives in parasite infectivity or survival in the host. IPC was isolated from promastigotes of three *L. (V.) braziliensis* strains and analyzed by positive- and negative-ion ESI-MS. The major IPC ions were characterized as eicosasphinganine and eicosasphingosine. Negative-ion ESI-MS revealed IPC ion species at *m/z* 778.6 (d20:1/14:0), 780.6 (d20:0/14:0), 796.6 (t20:0/14:0), 806.6 (d20:1/16:0), and 808.6 (d20:0/16:0). IPCs isolated from *L. (V.) braziliensis* and *L. (L.) major* showed significant differences in IPC ceramide composition. The major IPC ion from *L. (L.) major*, detected in negative-ion ESI-MS at *m/z* 780.6, was composed of ceramide d16:1/18:0. Our results suggest that sphingosine synthase (also known as serine palmitoyltransferase; SPT) in *L. (V.) braziliensis* is responsible for synthesis of a long-chain base of 20 carbons (d20), whereas SPT in *L. (L.) major* synthesizes a 16-carbon long-chain base (d16). A phylogenetic tree based on SPT proteins was constructed by analysis of sequence homologies in species of the *Leishmania* and *Viannia* subgenera. Results indicate that SPT gene position in *L. (V.) braziliensi*s is completely separated from that of members of subgenus *Leishmania*, including *L. (L.) major, L. (L.) infantum*, and *L. (L.) mexicana*. Our findings clearly demonstrate sphingoid base differences between *L. (V.) braziliensis* and members of subgenus *Leishmania*, and are relevant to future development of more effective targeted anti-leishmaniasis drugs.

## Introduction

Leishmaniasis is a group of diseases caused by protozoan parasites of the genus *Leishmania*. The prevalence of leishmaniasis worldwide was recently estimated as 12 million cases, with ~1.5–2 million new cases per year (WHO, 2017). The genus *Leishmania* is divided into two subgenera, *Leishmania (Leishmania)* and *Leishmania (Viannia)*, species of which are responsible for various clinical pathologies (cutaneous, mucocutaneous, and visceral leishmaniasis) and related biological, molecular, and biochemical features (Shaw, 2011).

The metabolism of *Leishmania* sphingolipids (SLs) has received considerable research attention during the past three decades because of their biological relevance as structural cell membrane components, and as bioactive compounds involved in cell-cell recognition, cell adhesion, cell growth, and signal transduction (Hakomori, 2004; Merrill, 2011; Adada et al., 2014). Studies by several groups have revealed clear association between SLs (and the enzymes responsible for their degradation) and parasite infectivity and disease progression (Straus et al., 1993; Zhang et al., 2003, 2010; Denny and Smith, 2004; Denny et al., 2004; Tanaka et al., 2007; Pillai et al., 2012).

In mammals, the *de novo* biosynthetic pathway of SLs, and consequent ceramide production, begins in the endoplasmic reticulum (ER), with condensation by serine palmitoyltransferase (SPT) of L-serine and palmitoyl-CoA (16:0) to form 3-ketosphinganine, followed by reduction of this intermediate to produce sphinganine (dihydrosphingosine, which presents two hydroxy groups) in a reaction that involves NADPH (Merrill, 2011; Markham et al., 2013). Sphinganine is subsequently acylated by ceramide synthase (CerS) to form dihydroceramide in the presence of n-acyl-CoA, followed by production of ceramide catalyzed by a dihydroceramide desaturase (Mullen et al., 2012). Evolutionary divergence has been demonstrated in these steps of the *de novo* pathway, and depends on the SL class and on the organism. SLs in trypanosomatids and mammals are usually derived from long-chain bases (LCBs) that contain two hydroxyl groups, and are denoted by the letter “d” (di) followed by the number of carbons in the chain. SLs in plants and fungi are derived from dihydroxylated or trihydroxylated sphingoid bases, and are denoted respectively by the letter “d” or “t.” Trihydroxylated sphingoid bases in plants and fungi are later acylated to form phytoceramide, a precursor of inositol phosphorylceramides (IPCs) and glycosyl inositol phosphorylceramides (GIPCs) (Markham et al., 2013; Del Poeta et al., 2014).

In the SL biosynthetic pathway, ceramides or phytoceramides synthesized in the ER are transferred to the Golgi apparatus, where further modification of the primary hydroxyl group results in formation of complex SLs, including glycosphingolipids (GSLs), sphingomyelin (SM), IPCs, and GIPCs (Zhang and Beverley, 2010; Zhang et al., 2010; Cingolani et al., 2016). SM and GSLs are the major SLs in mammals, whereas IPC is the major SL in *Leishmania* (Kaneshiro et al., 1986; Zhang and Beverley, 2010; Zhang et al., 2010). In the parasitic protozoan *Trypanosoma brucei*, IPC is found only in procyclic forms (Guther et al., 2006; Fridberg et al., 2008), whereas in *T. cruzi* IPC is synthesized as a precursor of GIPCs (Bertello et al., 1995; Uhrig et al., 1996; Zhang et al., 2010). In fungi and plants, there are two routes for biosynthesis of neutral and phosphorylated SLs. Neutral GSLs are usually derived from LCBs containing two hydroxy groups, whereas fungal IPCs and GIPCs are usually derived from LCBs containing trihydroxy groups, also known as phytosphingosine. Fungal monohexosylceramides present additional ceramide modifications, such as methylation of sphingoid base and hydroxylation of fatty acid (Suzuki et al., 2008; Del Poeta et al., 2014; Guimarães et al., 2014). In *Leishmania*, IPC was first identified in *L. (L.) donovani* promastigotes (Kaneshiro et al., 1986), which express 2.1 × 10^8^ molecules/cell, accounting for ~10% of phospholipids. IPC was detected subsequently in other *Leishmania* species (Zhang et al., 2003; Denny et al., 2004; Hsu et al., 2007).

*Leishmania* SLs have been shown to be involved in parasite morphology, infectivity, differentiation, and vesicular trafficking (Zhang et al., 2003, 2007; Denny et al., 2004; Tanaka et al., 2007; Castro et al., 2013). We demonstrated in 2006 that IPCs in *L. (V.) braziliensis* promastigotes are present in fractions enriched in membrane microdomains resistant to non-ionic detergent at 4°C (Yoneyama et al., 2006). More recently, we showed that IPCs in these promastigotes are essential for completion of cytokinesis, and play a major role in cell proliferation (Castro et al., 2013). IPCs are potentially useful targets for antiparasitic drugs, because they play an essential role in parasite virulence and are not expressed in mammalian cells. Studies using mass spectrometry (MS) have shown that IPCs in *L. (L.) mexicana* (Singh et al., 1988) and *L. (L.) major* (Hsu et al., 2007) contain mainly sphingoid bases d16:1 and d16:0 with C18:0 fatty acid, whereas those in *L. (V.) braziliensis* contain mainly C14:0 fatty acid (Yoneyama et al., 2006).

In the present study, we used collision-induced dissociation (CID) tandem MS with electrospray ionization (ESI) in both negative-ion mode [M-H]^−^ and positive-ion mode [M+H]^+^ to characterize IPCs from *L. (V.) braziliensis*, one of the major etiologic agents of cutaneous and mucocutaneous leishmaniasis in South America. In cutaneous leishmaniasis, parasites spread from the skin to the naso-oropharyngeal mucosa. The causative factors for such mucosal dissemination and resulting mucosal disease are poorly understood. Effective systemic treatment of cutaneous leishmaniasis caused by *L. (V.) braziliensis* will presumably reduce the risk of mucosal disease development. We focused on characterization of IPCs from *L. (V.) braziliensis* mainly because these molecules, and enzymes involved in their specific biosynthetic pathways, are promising targets for anti-*Leishmania* drug development (Denny et al., 2006; Suzuki et al., 2008; Young et al., 2012).

## Materials and methods

### Compounds

Stock solutions of 2 mM myriocin (a SL synthesis inhibitor) (Sigma-Aldrich; St. Louis, MO, USA) were prepared in dimethyl sulfoxide and stored at −70°C for a maximum of 1 month.

### Parasite culture

Promastigotes of *L. (V.) braziliensis* strains MHOM/BR/1987/M11272, MHOM/BR/2001/BA778 (from Laboratório de Imunoparasitologia, Centro de Pesquisa Gonçalo Muniz, Fundação Oswaldo Cruz, Bahia, Brazil) and MHOM/BR/1975/M2903 were cultured at 23°C by several passages of log-phase parasites in Medium 199 supplemented with 10% heat-inactivated fetal calf serum (Thermo Fisher Scientific/ Life Technologies, Brazil), 2 mM L-glutamine (Sigma-Aldrich), 0.02 mg ml^−1^ bovine hemin, 100 U ml^−1^ penicillin, 100 μg ml^−1^ streptomycin, and 2% sterile male human urine (complete medium). The starting inoculum (0.4 × 10^7^ cells ml^−1^) consisted of parasites isolated from early stationary phase. Parasites were collected either in log-phase or after 48 h (stationary phase). *L. (V.) major* strain MRHO/SU/59/P (LV39) was also cultured at 23°C in complete Medium 199.

### Parasite infectivity

*Leishmania (Viannia) braziliensis* MHOM/BR/2001/BA778 promastigotes were cultured (starting inoculum 0.4 × 10^7^ cells ml^−1^) in complete medium in the presence of myriocin (5 μM) or equivalent vehicle concentration for 6 days. Cell growth was estimated by counting cells with a hemocytometer (Improved Double Neubauer). Parasite viability was determined by SYTOX® Blue staining (Thermo Fisher/ Life Technologies; Carlsbad, CA, USA) as per the manufacturer's protocol, and shown to be >85% in control and myriocin-treated cultures. Parasite cultures were washed with PBS, cells were resuspended in PBS, and 1 × 10^6^ parasites (0.05 ml) were inoculated subcutaneously in footpads of female golden hamsters (*Mesocricetus auratus*) (groups of four). Eight weeks after inoculation (infection), inguinal lymph nodes were removed and homogenized in 3 ml culture medium. Parasite suspension from each node was plated in complete Medium 199, and parasites were quantified by limiting dilution in 96-well plates containing 120 μl well^−1^. Plates were kept at 23°C for 7 days. Parasite number (PN) per lymph node was estimated based on the highest dilution at which parasites were detected after 7 days (Lima et al., 1997), i.e., PN = highest dilution × 0.12 (ml well^−1^) × 3 (ml inguinal lymph node suspension). Experiments were performed in triplicate. All animal procedures were conducted in accordance with the recommendations of the Guide for the Care and Use of Laboratory Animals of the Brazilian National Council of Animal Experimentation (www.cobea.org.br). The protocol was approved by the Research Ethical Committee of Federal University of São Paulo (Comite de Etica em Pesquisa da Universidade Federal de Sao Paulo/Hospital Sao Paulo, Id# CEP 0226/10).

### Indirect immunofluorescence

Parasites were fixed with 2% formaldehyde in 10 mM phosphate buffer, pH 7.2, containing 150 mM NaCl (PBS), for 10 min. Some fixed cells were permeabilized with 0.1% saponin. Cells were washed, resuspended in 1 ml PBS, and 100 μl of the solution was added to coverslips pretreated with 0.1% poly-L-lysine. Coverslips were blocked for 3 h with 0.1% gelatin in PBS and for 1 h with 10% skimmed milk and 1% bovine serum albumin (BSA) in PBS. Parasites were incubated sequentially with primary antibody LST-1 (mouse IgM; reacts with *Leishmania* IPC; Godoy et al., MS in prep) and SST-1 (IgG3; reacts with *L. braziliensis* promastigote glycolipids) (Silveira et al., 2005) for 1 h, washed with PBS, and incubated with goat anti-mouse IgM (μ chain) conjugated to Alexa Fluor 647 or with goat anti-mouse IgG (gamma chain) conjugated to Alexa Fluor 488 (Thermo Fisher) in a solution containing 1% BSA and 0.01 mM 4,6-diamidino-2-phenylindole (DAPI) (Sigma-Aldrich) in PBS. Slides were examined under conventional or confocal fluorescence microscopy with a Leica SP5 TS system with a 100x/1.44 oil objective. Images were acquired under optimal instrument settings and processed by the ImageJ program (http://rsbweb.nih.gov/ij/).

### IPC purification

Lipids were extracted from ~2 × 10^8^ promastigotes isolated from either log phase or 48 h after stationary phase by homogenization with isopropanol/hexane/water (IHW) (55:20:25, v/v/v) and with chloroform/methanol (CM) (2:1, v/v) (Silveira et al., 2005). Supernatants were collected and dried by rotary evaporation. Samples were deacylated by alkaline hydrolysis with methylamine, and lipid extracts were resuspended in 1.0 ml methanol/ methylamine 30%/n-butanol (4:3:1, v/v/v) and incubated 3 h at 55°C in a dry bath (Serunian et al., 1989). For purification of IPC, the lipid fraction was resuspended in CM/ water (30:60:8, v/v/v; “solvent A”) and applied to DEAE-Sephadex A-25 columns. Columns were washed with five volumes of solvent A and then with five volumes of methanol, and IPCs were eluted with five volumes of 0.05 M sodium acetate in methanol (Toledo et al., 1995). The acidic fraction was dried, dialyzed exhaustively against distilled water, and partitioned with one volume of n-butanol saturated with water. The butanolic phase was collected and analyzed by HPTLC (Merck) and ESI-MS. IPCs were visualized on HPTLC as blue spots using Dittmer-Lester reagent (Yoneyama et al., 2006).

### ESI-MS

MS measurements were obtained using a triple-quadrupole instrument (model 310, Varian Inc./Agilent Technologies) with ESI source. Data acquisition was performed using the Varian MS Workstation program, V. 6.9. Sample analysis was conducted in positive-ion and negative-ion ESI modes with respective needle voltages 5.8 and 5 kV (Guan et al., 2010). Non-targeted mass scanning range was *m/z* 500–950, with capillary temperature 200°C, drying gas pressure (N_2_) 20 psi, and nebulizing gas (N_2_) 40 psi. Each MS2 individual ion fragmentation was optimized with regard to capillary and collision energy to minimize variations in relative ion abundance resulting from differences in dissociation rates. The CID gas was argon at 2 mTorr. The inlet system consisted of a direct infusion pump (Harvard Apparatus), and methanol/ water (8:2, v/v) with 5 mM ammonium formate as mobile phase, flow rate 30 μl min^−1^. Precursor ion scan (PREIS) for detection of ion containing phosphoinositol derivative *m/z* 259 (inositol monophosphate anion) and *m/z* 241 (inositol-1,2-cyclic phosphate anion) was performed in negative ion mode with capillary energy -110 V and collision energy 40 V. Typically, 20 scans were used for accumulation of non-targeted scan range, and 30 scans for analysis of precursor ions. Ion characterization was performed by CID in negative and positive ion mode with collision energy 15, 30, or 45 V (Hsu et al., 2007).

### Phylogenetic inference of serine palmitoyltransferase (SPT) proteins

Phylogenetic analysis was performed using gene data from SPT enzymes from many distinct species of the family Trypanosomatidae: *L*. (*L*.) *infantum, L*. (*L*.) *donovani, L*. (*L*.) *mexicana, L*. (*L*.) *major, L*. (*V*.) *braziliensis, L*. (*V*.) *guyanensis, L*. (*V*.) *panamensis, T*. *brucei*, and *T*. *cruzi* CL Brener. Sequences were chosen by homology search using BLAST software with annotated yeast and/or human proteins as baits. Sequences were retrieved from GenBank or GeneDB (TriTrypDB) (Table 1). ID sequences used to construct the tree are shown in Table 1. Yeast and human proteins were used as outgroup. Putative proteins were aligned using the Sea View software program (V. 3.2) with an embedded MUSCLE (Multiple Sequence Comparison by Log-Expectation) program. Alignment was performed using default parameters. The phylogenetic tree was inferred by Bayesian methods using the MrBayes software program (V. 3.2.3) with tree parameter optimization during generations. Bayesian inference was made by placing the phylogenetic root halfway between the two most divergent/ distant proteins (midpoint rooting). Data were saved every 100 generations and run in four chains and two runs. A Bayesian tree was inferred based on 1 × 10^7^ generations with burn-in = 75,000. Consensus trees were used to determine posterior probability values. The generated consensus tree was visualized using the Figtree software program (V. 1.4.2; http://tree.bio.ed.ac.uk/software/figtree/).

**Table 1:** Proteins and GenBank/GeneDB accessions used for phylogenetic studies.

**Species**	**Serine palmitoyl transferase** **SPT (LCB)**	**GenBank/GeneDB accession ID**
**Leishmania subgenus** *L. (L.) infantum*, *L. (L.) mexicana*, *L. (L.) major*	SPT (*L. major*: whole, N and catalytic)	LINJ_35_0320 LMXM_34_0320Lm jF.35.0320
**Viannia subgenus** *L. (V.) braziliensis*, *L. (V.) guyanensis*, *L. (V.) panamensis*	SPT (*L. braziliensis*: whole, N and catalytic)	LbrM.34.0360 CCM18812.1 LPMP_340300
*Trypanosoma brucei*	SPT	Tb10.70.3220
*Trypanosoma cruzi*	SPT	TcCLB.503453.100
Yeast (*Saccharomyces cerevisiae*)	LCB1 LCB2	AJS85732.1 NP_010347.1
Human	SPT1, SPT2, SPT3	NP_006406.1 NP_004854.1 NP_060797.2

### Data analysis

Quantitative data were analyzed by Student's *t*-test, using GraphPad Prism software program V. 5.0 (San Diego, CA, USA). Differences between means were considered statistically significant for *p* < 0.01. All experiments were performed in triplicate.

## Results

### Immunolocalization of *L. (V.) braziliensis* IPCs

**Figure 1:**
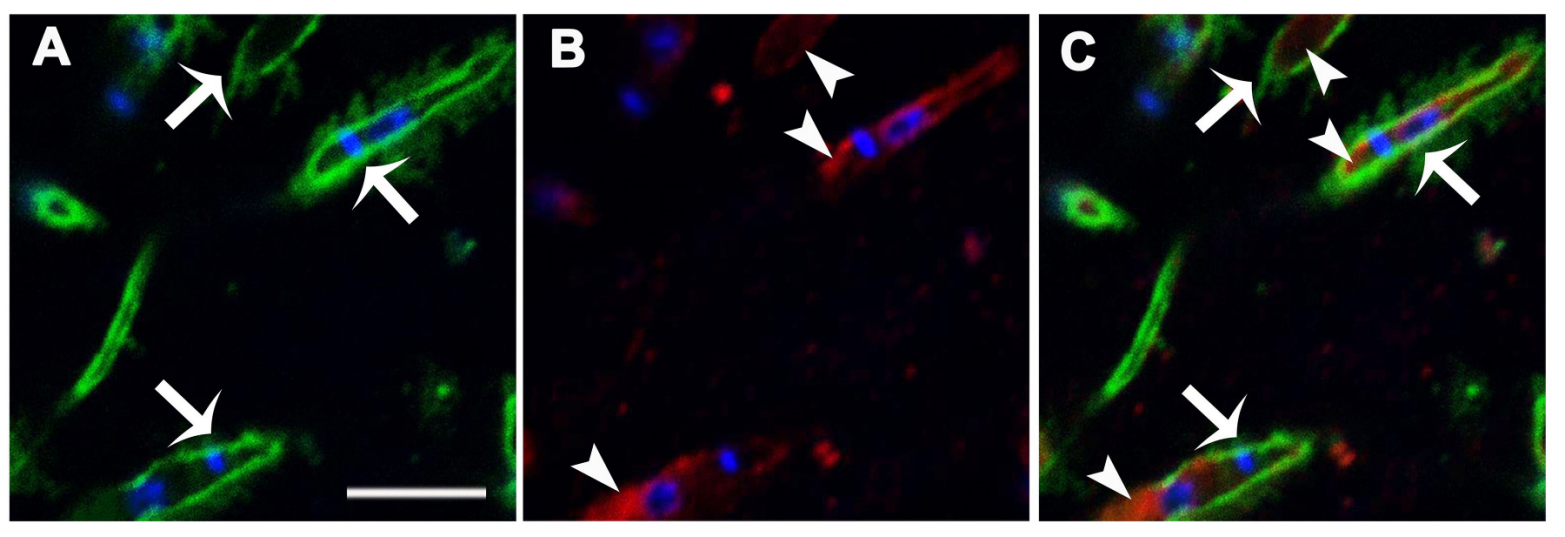
Immunolocalization of *L. (V.) braziliensis* (BA778) IPCs and GIPLs. Parasites were permeabilized with saponin 0.1%, fixed, labeled with mAb LST-1 (directed to *Leishmania* IPC; red), mAb SST-1 (directed to *L. (V.) braziliensis* glycolipids; green), and DAPI (nuclei and kinetoplasts; blue), and analyzed by confocal microscopy. **(A–C)** Representative optical section of focal plane showing intracellular labeling of IPC (arrowheads), and surface labeling of SST-1 (arrows). Scale bar: 5 μm.

To determine whether IPCs are expressed on the *Leishmania* surface, parasites were double-labeled with monoclonal antibodies (mAbs) directed to *L. (V.) braziliensis* glycolipids (mAb SST-1), and to *Leishmania* IPC (mAb LST-1). Representative images from a z-stack confocal series are shown in Figure 1. SST-1 (green) labels the parasite surface (Figure 1A; arrows), and LST-1 (red) labels internal fluorescence (Figure 1B; arrowheads). No co-localization was observed between SST-1 and LST-1 (Figure 1C). These findings indicate that no IPCs are localized on the parasite surface. An animation of 29 confocal z-stack slices (Video 1, Supplemental Information) shows differing focal planes of parasites labeled with mAb LST-1 (red), mAb SST-1 (green), and DAPI (blue).

### Effect of myriocin on *L. (V.) braziliensis* infectivity

**Figure 2:**
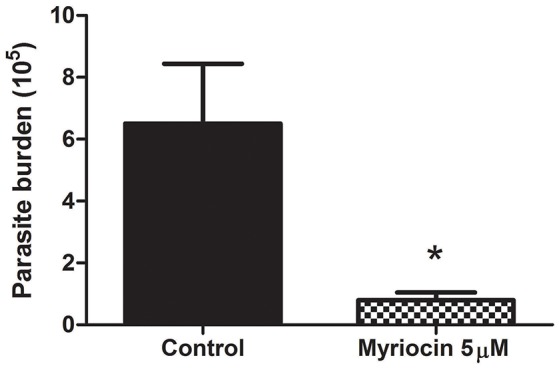
Parasite burden of inguinal lymph nodes of golden hamsters infected subcutaneously via footpad with stationary growth phase of *L. (V.) braziliensis* (BA778) promastigotes pretreated (or not) with 5 μM myriocin. Nodes were removed after 8 weeks of infection, and parasite burden was determined by limiting dilution as described in Materials and Methods. ^*^p < 0.05.

We investigated the effect of myriocin on parasite infectivity, in view of our previous observations that 5 μM myriocin reduces *L. (V.) braziliensis* IPC expression, growth rate, and cytokinesis (Castro et al., 2013). Golden hamsters were infected subcutaneously via footpad with promastigotes treated (or not) with 5 μM myriocin for 6 days. After 8 weeks, infection rate was determined in inguinal lymph nodes by limiting dilution assay. PN per lymph node was significantly reduced (8-fold) for myriocin-treated parasites in comparison with controls (Figure 2), suggesting that sphingosine derivatives (e.g., IPCs or biosynthetic intermediates) play an important role in host cell infection.

### Characterization of *L. (V.) braziliensis* IPCs

**Figure 3:**
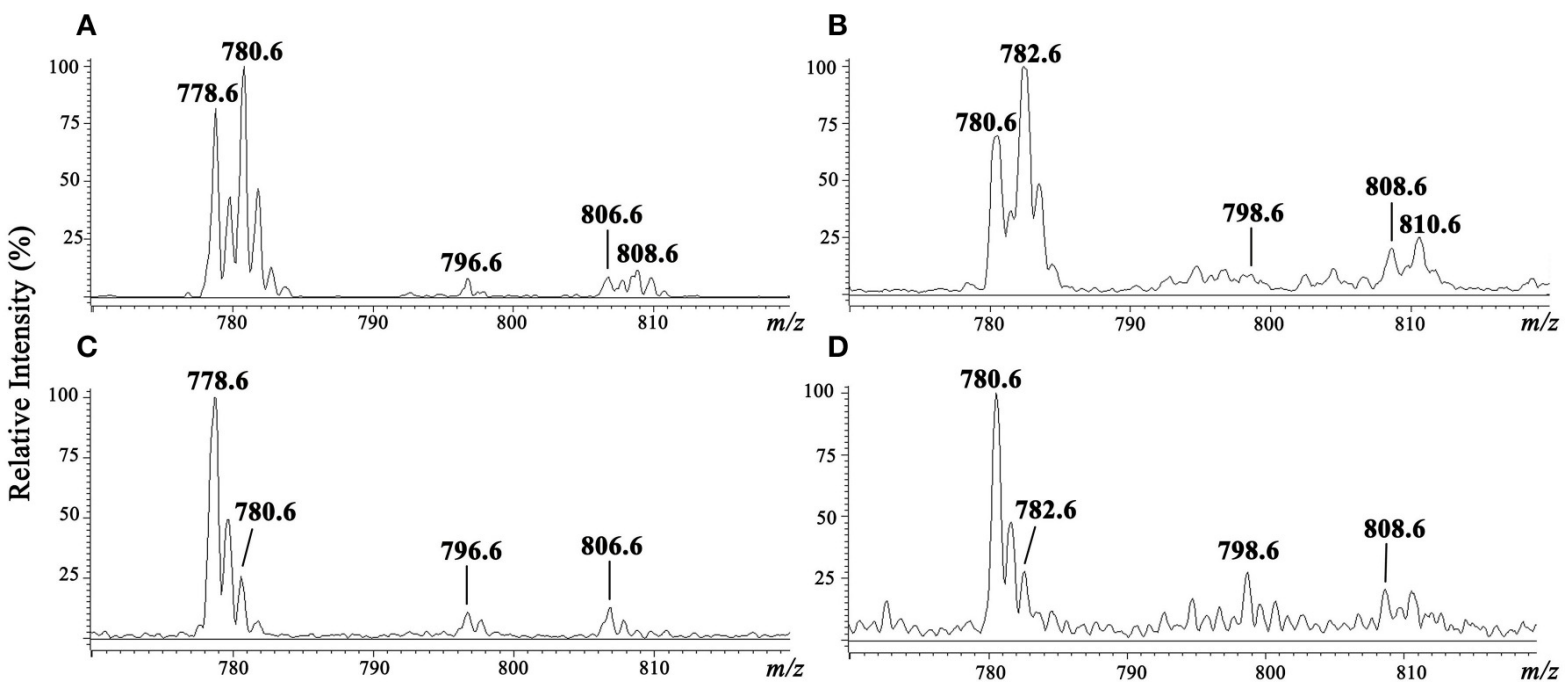
ESI-MS spectra of [M-H]^−^ and [M+H]^+^ ions of IPC purified from *L. (V.) braziliensis* (M11272) and *L. (L.) major* (stationary growth phase). **(A,C)** ESI-MS spectra of [M-H]^−^ ion of IPC fractions of *L. (V.) braziliensis* and *L. (L.) major*, respectively. **(B,D)** ESI-MS spectra of [M+H]^+^ ion of IPC fractions of *L. (V.) braziliensis* and *L. (L.) major*, respectively.

Purified IPC fractions from parasites were characterized by ESI-MS. Because IPC has a phosphate residue that is readily ionizable, ESI-MS in negative ion mode was the appropriate method for quick detection of IPC ions. All IPC ions were characterized by detection of inositol phosphate fragments at *m/z* 259, corresponding to inositol monophosphate anion (InsP), and at *m/z* 241, corresponding to inositol-1,2-cyclic phosphate anion (InsP-H_2_O). ESI-MS of [M-H]^−^ ions of IPC fractions purified from three *L. (V.) braziliensis* strains (BA778, M2902, M11272) were performed, and similar spectral profiles were observed for all strains. In full scan of IPC fraction from negative-ion ESI-MS of *L. (V.) braziliensis* (M11272), major ions were detected at *m/z* 778.6 and 780.6, and minor components were detected at *m/z* 796.6, 806.6, and 808.6 (Figure 3A). ESI-MS of [M-H]^−^ ions of *L. (L.) major* IPC fraction showed a distinctive profile: the major IPC ion was at *m/z* 778.6, relative abundance of *m/z* 780.6 was significantly reduced, and minor IPC ions were detected at *m/z* 796.6 and 806.6 (Figure 3C). In full scan spectra of IPC fractions in positive-ion mode ESI-MS, corresponding protonated ions were detected at *m/z* 780.6, 782.6, 798.6, 808.6, and 810.6 for *L. (V.) braziliensis*, and at *m/z* 780.6, 782.6, 798.6, and 808.6 for *L. (L.) major* (Figures 3B,D).

### Characterization by ESI-MS/MS of *L. (V.) braziliensis* IPC [M-H]^−^ ions at m/z 778.6 and 780.6, and of [M+H]^+^ ions at m/z 780.6 and 782.6

To characterize the structures of individual IPC ions, CID was performed in negative ion mode for confirmation of inositol derivative ions, and in positive ion mode for characterization of ceramide moiety. LCB ions give rise to well-established ion signatures comprised of double dehydrated ions, e.g., *m/z* 292, 294, 236, and 238 correspond respectively to d20:1-, d20:0-, d16:1-, and d16:0-LCB.

**Figure 4:**
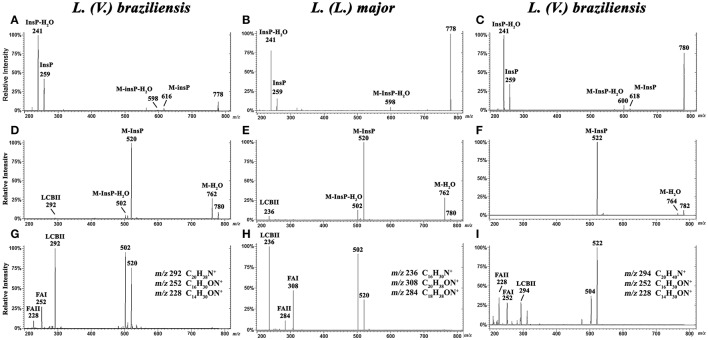
ESI-CID-MS/MS spectra of IPC fractions obtained from *L. (V.) braziliensis* (M11272) and *L. (L.) major* at stationary phase. **(A,B)** Negative-ion ESI-CID-MS/MS spectra, *m/z* 778.6, from *L. (V.) braziliensis* and *L. (L.) major*, with collision energy 45 and 30 V, respectively. **(C)** Negative-ion ESI-CID-MS/MS spectra of *L. (V.) braziliensis, m/z* 780.6, with collision energy 30 V. **(D,E)** Positive-ion ESI-CID-MS/MS spectra, *m/z* 780.6, from *L. (V.) braziliensis* (d20:1/14:0) and *L. (L.) major* (d16:1/18:0), with collision energy 15 V. **(F)** Positive-ion ESI-CID-MS/MS spectra of *L. (V.) braziliensis, m/z* 782.6 (d20:0/14:0), with collision energy 15 V. **(G,H)** Positive-ion ESI-CID-MS/MS spectra, *m/z* 780.6, from *L. (V.) braziliensis* (d20:1/14:0) and *L. (L.) major* (d16:1/18:0), respectively, with collision energy 30 V. **(I)** Positive-ion ESI-CID-MS/MS spectra of *L. (V.) braziliensis, m/z* 782.6 (d20:0:1/14:0), with collision energy 30 V.

CID was performed for major IPC ions detected in *L. (V.) braziliensis* and *L. (L.) major* (Hsu et al., 2007) by ESI-MS/MS in negative-ion mode (Figures 4A–C) and positive-ion mode (Figures 4D–I). By analysis of tandem MS product ions of corresponding [M-H]^−^ ions at *m/z* 778.6 from *L. (V.) braziliensis* and *L. (L.) major* (Figures 4A,B) and at *m/z* 780.6 from *L. (V.) braziliensis* (Figure 4C), phosphoinositol ion signatures were confirmed by the presence of ions at *m/z* 259 (InsP) and 241 (InsP-H_2_O).

Ceramide moieties were characterized by CID in positive-ion mode using various collision energies. MS/MS product ion spectra of *L. (V.) braziliensis* [M+H]^+^ ions at *m/z* 780.6, using collision energy of 15V, yielded expected fragments at *m/z* 762 (M-H_2_O), 520 (M-InsP), and 502 (M-InsP-H_2_O) (Figure 4D). We also detected a fragment at *m/z* 292, the signature ion of d20:1-LCB (LCB product after elimination of one water molecule). Application of higher collision energy (45 V) for the ion at *m/z* 780.6 (Figure 4G), in addition to fragments at *m/z* 520, 502, and 292, led to detection of spectral signature ions at *m/z* 252 and 228, corresponding to a 14:0-fatty acid acyl substituent (FAI and II derivatives C_16_H_30_ON^+^ and C_14_H_30_ON^+^, respectively), suggesting that this compound was d20:1/14:0-IPC. Figure S1, at Supplemental Information, shows the structures of main MS/MS products of *L. (V.) braziliensis* [M+H]^+^ ion at *m/z* 780.6.

ESI-MS/MS spectra of *L. (L.) major* [M+H]^+^ ion at *m/z* 780.6, using collision energies 15 and 30V, gave rise as expected to ions at *m/z* 762 (M-H_2_O), 520 (M-InsP), and 502 (M-InsP-H_2_O) (Figures 4E,H), as observed previously for *L. (V.) braziliensis*. The ion profile, in terms of LCB and fatty acid composition, was distinct from that of *L. (V.) braziliensis*. Ions were detected at *m/z* 236 (indicating the presence of d16:1 sphingoid base), and fragments at *m/z* 308 and 284 (FAI and II derivatives C_20_H_38_ON^+^ and C_18_H_38_ON^+^, respectively), identifying the 18:0-fatty acyl substituent, as described by Hsu et al. (2007).

ESI-MS/MS spectra of *L. (V.) braziliensi*s [M+H]^+^ ion at *m/z* 782.6 using collision energies 15 and 45 V (Figures 4F,I) generated fragments at *m/z* 764 (M-H_2_O), 522 **(**M-InsP**)**, 504 (M-InsP-H_2_O), 294 (spectral signature of d20:0-LCB), 252, and 228 (spectral signatures of 14:0-fatty acid acyl substituent) (FAI and II derivatives, C_16_H_30_ON^+^ and C_14_H_30_ON^+^, respectively), indicating that this compound is d20:0/14:0-IPC.

### Characterization by ESI-MS/MS of *L. (V.) braziliensis* IPC [M-H]^−^ ions at m/z 796.6, 806.6, and 808.6, and of [M+H]^+^ ions at m/z 798.6, 808.6, and 810.6

**Figure 5:**
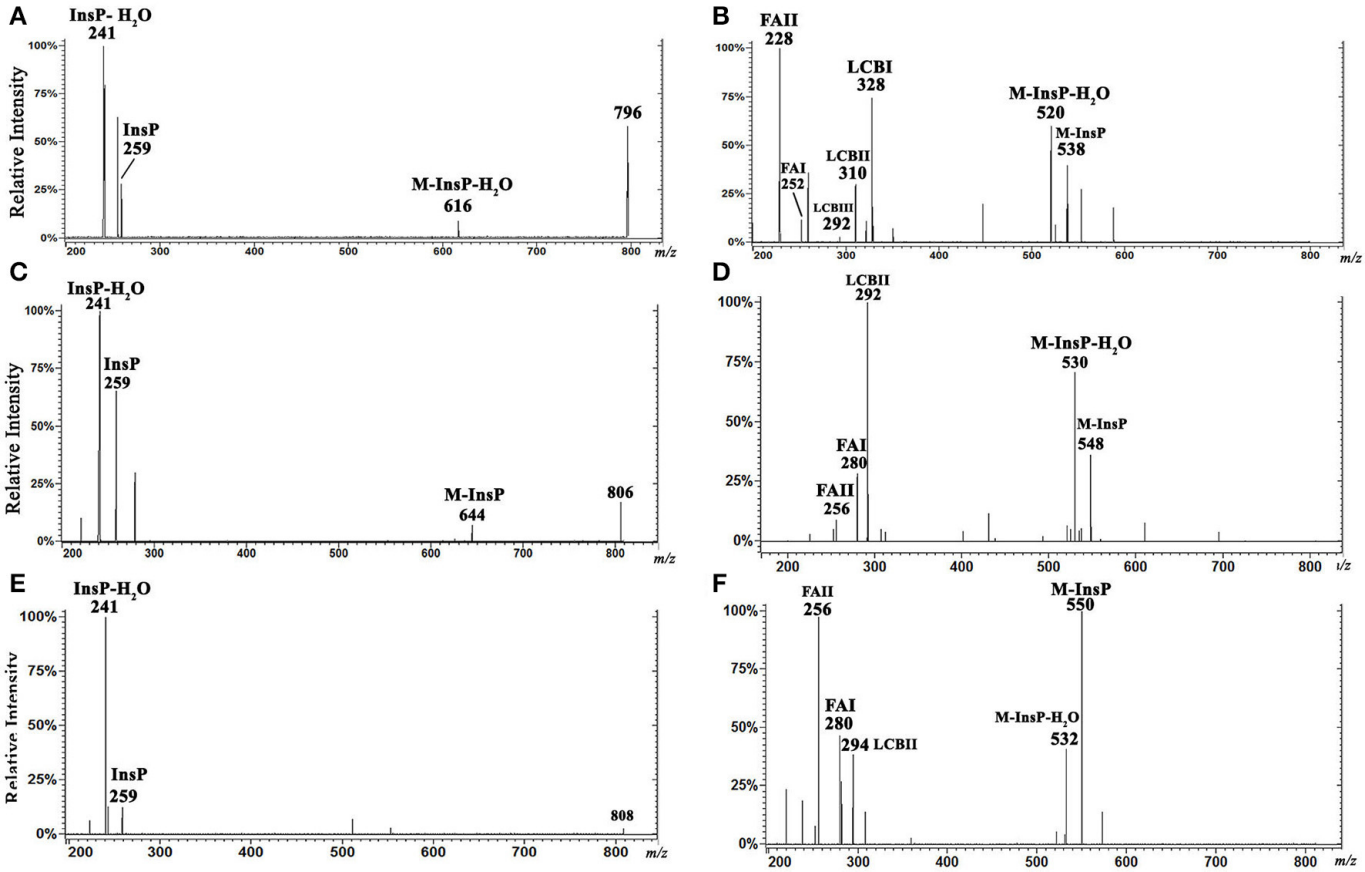
ESI-CID-MS/MS spectra of IPC fractions obtained from *L. (V.) braziliensis* (M11272) stationary growth phase. **(A,C,E)** Spectra of *m/z* 796.6 (t20:0/14:0), 806.6 (d20:1/16:0), and 808.6 (d20:0/16:0), respectively, in negative-ion mode with collision energy 15 V. **(B,D,F)** Spectra of *m/z* 798.6 (t20:0/14:0), 808.6 (d20:1/16:0), and 810.6 (d20:0/16:0), respectively, in positive-ion mode with collision energy 5 V.

We also characterized minor IPC ions of *L. (V.) braziliensis*. ESI-MS/MS spectra of [M-H]^−^ ions at *m/z* 796.6, 806.6, and 808.6 are shown in Figures 5A,C,E. Inositol phosphate signature ions at *m/z* 259 (InsP) and 241 (InsP-H_2_O) were detected in all IPC ions.

Analysis of ESI-MS/MS spectra of the [M+H]^+^ ion at *m/z* 798.6 allowed us to characterize the ceramide moiety. Using collision energy 45V (Figure 5B), the spectra gave rise to ions at *m/z* 538 (M-InsP), 520 (M-InsP-H_2_O), 328, 310, 292 (characteristic products of phytosphingosine t20:0), 252, and 228 (spectral signatures of 14:0 fatty acid acyl substituent) (FAI and II derivatives C_16_H_30_ON^+^ and C_14_H_30_ON^+^, respectively). These findings suggest that this ion (*m/z* 798.6 in positive-ion mode) corresponds to t20:0/14:0-IPC.

Analysis of ESI-MS/MS spectra of the [M+H]^+^ ion at *m/z* 808.6 using collision energy 45 V (Figure 5D) generated ions at *m/z* 548 (M-InsP), 530 (M-InsP-H_2_O), 292 (characteristic of sphingoid base d20:1), 280, and 256 (corresponding to fatty acid 16:0 acyl substituent) (FAI and II derivatives C_18_H_34_ON^+^ and C_16_H_34_ON^+^, respectively), suggesting that this compound is d20:1/16:0-IPC.

Analysis of ESI-MS/MS spectra of the [M+H]^+^ ion at *m/z* 810.6 using collision energy 45 V (Figure 5F) generated ions at *m/z* 550 (M-InsP), 532 (M-InsP-H_2_O), 294 (characteristic of d20:0), 280, and 256 (corresponding to fatty acid 16:0 acyl substituent) (FAI and II derivatives C_18_H_34_ON^+^ and C_16_H_34_ON^+^, respectively), suggesting that this compound is d20:0/16:0-IPC.

IPC proposed structures and positive- and negative-ion ESI-CID-MS/MS fragments of IPC ions from *L. (V.) braziliensis* are summarized in Table 2. In contrast to IPCs of *L. major* (Hsu et al., 2007), those of *L. (V.) braziliensis* display predominantly 20-carbon LCBs (d20:1, d20:0, t20:0), and C14:0 and C16:0 fatty acids. Positive-ion ESI-CID-MS/MS of IPC ions from *L. (V.) braziliensis* did not detect product ions related to d16:0 (*m/z* 238) or d16:1(*m/z* 236). Positive-ion ESI-CID-MS/MS of *L. (L.) major* IPC ion at *m/z* 780.6 did not detect sphingoid base product related to d20:1 (*m/z* 292) (Figure 4H, Table 2). The same ESI-MS/MS fragment profile was observed for IPC isolated from *L. (V.) braziliensis* strains M11272, BA778, and M2903.

**Table 2:** *Leishmania (V.) braziliensis* IPC-related product ions (*m/z*) formed in negative- and positive ESI-MS/MS.

***L. (V.) braziliensis***											L. (L.) major^*^	
**Sphingoid** **Fatty acid**	**d20:1** **14:0**		**d20:0** **14:0**		**t20:0** **14:0**		**d20:1** **16:0**		**d20:0** **16:0**		**d16:0** **18:0**	
	[M-H]^−^	[M+H]^+^	[M-H]^−^	[M+H]^+^	[M-H]^−^	[M+H]^+^	[M-H]^−^	[M+H]^+^	[M-H]^−^	[M+H]^+^	[M-H]^−^	[M+H]^+^
M	778	780	780	782	796	798	806	808	808	810	778	780
M-Ins	616		618				644					
M-Ins-H_2_O	598		600		616						598	
InsP	259		259		259		259		259		259	
InsP-H_2_O	241		241		241		241		241		241	
M-H_2_O		762		764								762
M-InsP		520		522		538		548		550		520
M-InsP-H_2_O		502		504		520		530		532		502
LCBI product						328						
LCBII product		292		294		310		292		294		236
LCBIII product						292						
FAI derivative		252^c^		252		252		280^e^		280		308^a^
FAII derivative		228^d^		228		228		256^f^		256		284^b^

M, ion mass; Ins, inositol; InsP, inositol phosphate; LCBI, LCBII, and LCBIII products correspond respectively to LCB fragment, LCB fragment–H_2_O and LCB fragment–2H_2_O; FA, fatty acid derivative.

* Major IPC ion detected in Leishmania (L.) major.

ab Fatty acid derivatives described by Hsu et al. (2007) as C_20_H_38_ON^+^, and C_18_H_38_ON^+^, respectively.

c C_16_H_30_ON^+^.

d C_14_H_30_ON^+^.

e C_18_H_34_ON^+^.

f C_16_H_34_ON^+^.

### Phylogenetic inference of SPT proteins

**Figure 6:**
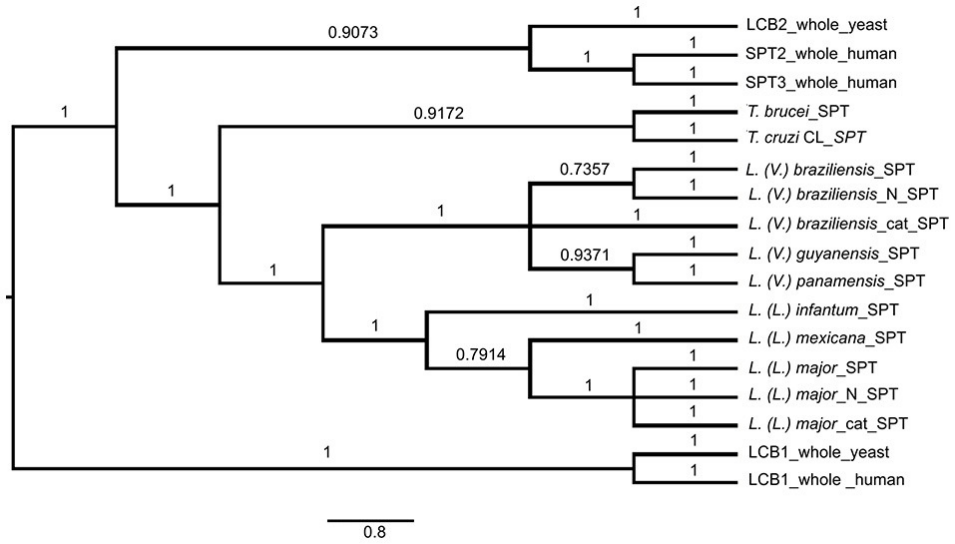
Bayesian consensus phylogeny of SPT proteins. The phylogram represents a consensus of 13 SPT sequences. The root was inferred using midpoint rooting. Posterior probabilities exceeding 0.5 are shown in the branches. The tree topology indicates that SPTs of members of *Leishmania* subgenus *Leishmania* are separated from those of *Leishmania* subgenus *Viannia*, and that SPTs of *Leishmania* species are separated from those of *Trypanosoma brucei* and *T. cruzi*. The scale of the generated tree (see 0.8 bar) represents the number of substitutions per sequence position. Accession numbers of SPT sequences are shown in Table 1. N, N-terminal end of protein sequence; cat, catalytic region of protein sequence.

SPT in *L. (V.) braziliensis* is responsible for synthesis of 20-carbon LCB (d20), whereas *L. (L.) major* presents mainly 16-carbon LCB (d16), and *T. brucei* and *T. cruzi* SLs present mainly 18-carbon sphingoid bases (d18) (Uhrig et al., 1996; Richmond et al., 2010; Vacchina et al., 2012). We therefore considered the possibility that these differences are related to SPT sequence homology with either *T. cruzi* or *T. brucei*. We used Bayesian inference to identify clades composed of species-related proteins (Figure 6). The SPT protein sequences belonged to different clades, presenting specific SPT nodes and branches among trypanosomatid species. Human and yeast LCB enzymes were more closely related to each other, and were totally separated from *T. brucei, T. cruzi*, and *Leishmania* enzymes. *Leishmania* SPT was subdivided into two subgroups. The species of the *Viannia* subgenus *(L. (V.) braziliensis, L. (V.) guyanensis* and *L. (V.) panamensis*) were completely separated from those of the *Leishmania* subgenus (*L. (L.) infantum, L. (L.) mexicana*, and *L. (L.) major*), and comprised two distinct clades. SPTs of *T. brucei* and *T. cruzi* also represented different clades.

In addition to whole-protein sequences of *Leishmania* SPTs, we also analyzed N-terminal and catalytic portions of SPTs of *L. (V.) braziliensis* and *L. (L.) major* to determine whether sequence homologies refer specifically to the catalytic portion. Phylogenetic results were similar for sequences of SPT whole proteins, N-terminal regions, and catalytic regions (Figure 6), indicating that SPT catalytic regions of *Leishmania* are not closer or more similar to those of either humans or trypanosomes.

## Discussion

IPCs are the predominant SLs in the genus *Leishmania* (Zhang and Beverley, 2010), including the medically important species *L. (V.) braziliensis* (Yoneyama et al., 2006). Our confocal microscopy studies using mAb LST-1 (directed to *Leishmania* IPC) showed that IPCs and GIPLs are not co-localized in *L. (V.) braziliensis* promastigotes; GIPLs are found on the parasite surface whereas IPCs are localized internally. Labeling of IPCs was observed only when parasites were permeabilized with 0.1% saponin, confirming the internal localization of IPCs. Previous studies have demonstrated the involvement of *Leishmania* SLs in a variety of biological processes, including differentiation, replication, trafficking, and virulence (Zhang et al., 2010). We demonstrated previously that *L. (V.) braziliensis* promastigotes treated with the SPT inhibitor myriocin displayed reduced IPC accumulation and incomplete cytokinesis (Castro et al., 2013).

In the present study, we investigated the role of SLs in *L. (V.) braziliensis* infectivity. Golden hamsters were infected by subcutaneous footpad injection of promastigotes treated (or not) with 5 μM myriocin for 6 days. After 8 weeks of infection, PN was counted in inguinal lymph nodes, and found to be ~8-fold lower for myriocin-treated parasites in comparison with controls. Our findings suggest that IPCs (and/or their intermediate SLs) are important for *L. (V.) braziliensis* infectivity, for their survival during the first few hours inside macrophages, for differentiation to amastigotes, and for amastigote proliferation. We obtained similar results in a previous study of *L. (L.) amazonensis* mouse experimental infection, in which parasite burden was reduced by infection of mouse footpads with promastigotes pretreated with a different SL inhibitor: the IPC synthase inhibitor aureobasidin A (Tanaka et al., 2007). Along the same line, an SPT subunit 2 knockout mutant of *L. (L.) major* displayed defective membrane trafficking events in extracellular promastigotes, thus delaying promastigote infection in BALB/c mice (Denny et al., 2004). In this paper we demonstrated that IPC is present intracellularly in *leishmania*, and as showed previously IPC is concentrated in non-ionic detergent insoluble domains (Yoneyama et al., 2006), the above findings and others suggest that synthesis of SLs in parasites, and the final biosynthetic product (IPC), are involved in vesicle formation, membrane trafficking, protein sorting, and cytokinesis, and play important roles in parasite growth/survival and hence in the establishment of infection. Thus, parasite SLs and the related biosynthetic enzymes are potentially useful targets in anti-parasite chemotherapeutic strategies. In a previous study, we used gas chromatography/MS to show that IPCs of *L. (V.) braziliensis* contain mainly C14:0 fatty acid (Yoneyama et al., 2006), but did not determine IPC mass or sphingoid bases. IPCs of *L. (L.) major* and *L. (L.) donovani* contain mainly C18:0 fatty acids (Hsu et al., 2007; Zheng et al., 2010). In the present study, we purified IPCs from three *L. (V.) braziliensis* strains, isolated from patients living in different regions of Brazil: reference strain MHOM/BR/1975/M2903 from a subject in Pará (Northern Brazil), strain MHOM/BR/01/BA788 from a cutaneous leishmaniasis patient in Bahia State (Northeastern Brazil), and strain MHOM/BR/1987/M11272 from a cutaneous leishmaniasis patient in Paraná State (Southern Brazil). IPC molecules were detected by negative-ion ESI-MS, as described by Hsu et al. (2007), who demonstrated that the detection threshold for [M-H]^−^ ion is 10 times lower than those for [M+H]^+^, [H+Li]^+^, and [M-H+Li]^+^ ions. A tandem MS approach using positive-ion MS/MS allowed us to determine the ceramide structure of IPCs and discriminate among isomeric structures, providing better understanding of the metabolic pathway of SLs in *L. (V.) braziliensis*, and to identify novel IPC structures. The major IPC ions detected by negative-ion ESI-MS were at *m/z* 778 and 780 for all three strains, regardless of whether ions were isolated at logarithmic or stationary growth phase. These two ions accounted for >70% of IPCs expressed in the parasites. In negative-ion mode, the full scan profile of IPC fractions isolated from the three strains showed ions at *m/z* 778.6, 780.6, 796.6, 806.6, and 808.6 (Figure 3A). Predominant ion at *m/z* 778.6 has also been described for IPCs purified from *L. (L.) major* and *L. (L.) donovani* (Hsu et al., 2007; Zheng et al., 2010). When ceramide moieties were characterized by positive-ion ESI-MS/MS, clear differences were detected in comparison with IPCs from *L. (L.) major* (Hsu et al., 2007) and *L. (L.) donovani* (Kaneshiro et al., 1986). *L. (V.) braziliensis* IPC [M+H]^+^ ions at *m/z* 780.6 and 782.6 displayed fragments at *m/z* 292 (20:1-LCB), 294 (20:0-LCB), and 228 (C14:0-fatty acyl substituent). IPC ions with the same mass in positive-ion mode (*m/z* 780 and 782) from *L. (L.) major* presented d16:1 and d16:0 LCBs, respectively, with C18:0-fatty acid. IPCs are the major SLs in *Leishmania*; these findings therefore suggest strongly that *L. (V.) braziliensi*s preferentially synthesizes sphingoid bases through condensation of L-serine and stearoyl-CoA (18:0) to produce d20:0 in LCBs and ceramide, whereas *L. (L.) major* and *L. (L.) donovani* (subgenus *Leishmania*) preferentially synthesize d16:0 sphingoid base through condensation of L-serine and myristoyl-CoA (14:0) (Kaneshiro et al., 1986; Hsu et al., 2007). In contrast, mammals preferentially express 18-carbon LCBs (d18:0 and d18:1) in ceramide, synthesized by SPT proteins (Pruett et al., 2008; Merrill, 2011). Phylogenetic data for SPT of *L. (V.) braziliensis* showed that the SPT protein sequence of *L. (V.) braziliensis* is completely separated from those of species in the *Leishmania* subgenus, suggesting that *L. (V.) braziliensis* and the *Leishmania* subgenus species represent different clades. The constructed trees suggest that genetic factors are involved in SPT separation. More precise studies are needed to clarify this issue. The two *Leishmania* subgenera may have differentiated at different times, as evidenced by the SPT protein data, and this separation may be responsible for observed differences in enzyme functions and specificities. The overall conclusion from the SPT phylogenetic tree is that neither *L. major* nor *L. braziliensis* is closer to *T. cruzi* or *T. brucei*. It appears that the common SPT ancestor of *L. braziliensis* and *L. major* separated from the Trypanosomatidae prior to the separation of *Leishmania*.

IPCs isolated from *L. (V.) braziliensis* vs. *L. major*, in addition to differences in LCBs, show distinctive fatty acid compositions. In *L. (V.) braziliensis* IPCs, the major fatty acid is C14:0, and there are only trace amounts of fatty acid 16:0, as detected in positive-ion mode ESI-MS/MS at *m/z* 808.6 and 810.6. In contrast, the major fatty acid in *L. (L.) major and L. (L.) donovani* IPCs is stearic acid (C18:0) (Hsu et al., 2007; Zheng et al., 2010). Our findings also suggest that different *Leishmania* subgenera have differing CerS substrates. Members of the *Viannia* subgenus, e.g., *L. (V.) braziliensis*, preferentially use d20:0 and myristoyl-CoA (14:0) or palmitoyl-CoA (16:0), whereas members of the *Leishmania* subgenus, e.g., *L. (L.) major* and *L. (L.) donovani*, preferentially use d16:0 and stearoyl-CoA (18:0) (Hsu et al., 2007; Zhang and Beverley, 2010; Zhang et al., 2010). Mammals, in contrast, preferentially use d18:0 and various acyl-CoAs (carbon chain length varying from 16 to 26 depending on the CerS; Levy and Futerman, 2010; Merrill, 2011).

The existence of *Leishmania*-specific IPCs suggests that these parasitic microorganisms have a complete and functional SL biosynthetic pathway (Denny et al., 2004; Zhang et al., 2010), for which LCB synthase, CerS, IPC activities, and/or IPC genes have been described (Zhang et al., 2003, 2007; Denny et al., 2004, 2006; Tanaka et al., 2007; Castro et al., 2013; Ramakrishnan et al., 2013; Mandlik et al., 2014). However, enzyme activities for *Leishmania* are not yet characterized, and so far are based on or inferred from the final SL biosynthetic product: IPC ceramide structure. In regard to other trypanosomatids, Figueiredo et al. (2012) reported that TcCerS in *T. cruzi* preferentially uses dihydrosphingosine base as sphingosine base and palmitoylCoA as substrate, and Sevova et al. (2010) showed that specific SL synthases in *T. brucei, T. cruzi*, and *L. major* transfer polar head groups from phosphoglycerolipid donors to generate SM, ethanolamine phosphorylceramide, and IPC using 6-((*N*-(7-nitrobenz-2-oxa-1,3-diazol-4-yl)amino)hexanoyl)sphingosine C_6_-ceramide.

Future studies on *Leishmania* enzyme specificities in LCB synthesis and concurrent ceramide synthesis will contribute to the development of more effective drugs having high ligand efficiency indices against *L. (V.) braziliensis, L. (L.) major*, or *L. (L.) donovani*, but low (or zero) ligand efficiency to mammalian host cells.

## Author contributions

ED and AS conceived and designed the experiments, and wrote the manuscript. ED performed the experiments. ED and MT performed mass spectrometric analysis. RW and DB performed phylogenetic studies. ED and RM performed confocal microscopy and image analysis. HT critically reviewed and revised the manuscript.

### Conflict of interest statement

The authors declare that the research was conducted in the absence of any commercial or financial relationships that could be construed as a potential conflict of interest.

## Abbreviations

BSA: bovine serum albumin
CerS: ceramide synthase
CID-MS: collision-induced dissociation mass spectrometry
CM: chloroform/methanol
ER: endoplasmic reticulum
ESI-MS: electrospray ionization mass spectrometry
GIPC: glycosyl inositol phosphorylceramide
HPTLC: high performance thin layer chromatography
IHW: isopropanol/hexane/water
IPC: inositol phosphorylceramide
LCB: long-chain base
*mAb*: *monoclonal antibody*
NLS: neutral loss scan
PBS: phosphate buffered saline
PC: phosphatidylcholine
PE: phosphatidylethanolamine
PI: phosphatidylinositol
PL: phospholipid
PREIS: Precursor íon scan
PS: phosphatidylserine
SL: sphingolipid
SM: sphingomyelin
SPT: serine palmitoyltransferase.
